# Supplementary material for: Protein Concentration Affects the Food Allergen γ-Conglutin Uptake and Bacteria-Induced Cytokine Production in Dendritic Cells
Source: Biomolecules. 2023 Oct 16;13(10):1531. doi: 10.3390/biom13101531 (PMC10605286; doi:10.3390/biom13101531)
Supplement: Supplementary file 1 [file biomolecules-13-01531-s001.zip › biomolecules-2560787-supplementary.pdf]

biomolecules

MDPI

Supplementary materials

Protein Concentration Affects the Food Allergen γ-Conglutin Uptake and Bacteria-Induced Cytokine Production in Dendritic Cells

Giuditta C. Heinzl [1,2], Danny Blichfeldt Eriksen [2], Peter Riber Johnsen [2], Alessio Scarafoni [1] and Hanne Frøkiær [2,*]

[1] Department of Food, Environmental and Nutritional Sciences (DeFENS), University of Milan, Via Celoria 2, 20133 Milan, Italy; giuditta.heinzl@unimi.it (G.C.H.); alessio.scarafoni@unimi.it (A.S.)
[2] Department of Veterinary and Animal Sciences, University of Copenhagen, Ridebanevej 9, 1871 Frederiksberg, Denmark; qlm234@alumni.ku.dk (D.B.E.); peterriber@sund.ku.dk (P.R.J.)
* Correspondence: hafr@sund.ku.dk

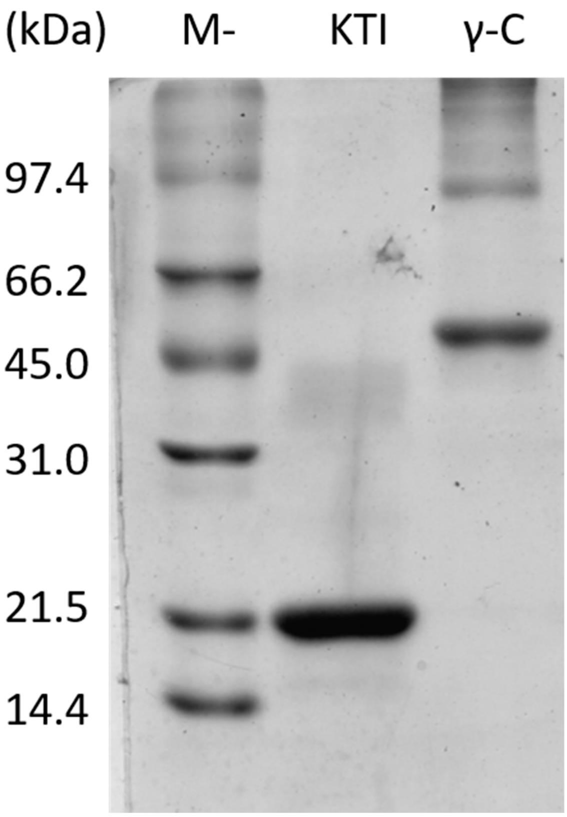

Supplementary Figure S1. SDS-PAGE of of KTI and γ-Conglutin proteins. Left lane: molecular marker, Middle lane: KTI and right lane: γ-Conglutin.

The γ-Conglutin bands correspond to the monomer (≈45 kDa) and dimer (≈90 kDa) and larger aggregates, respectively, as the native protein has been shown to undergo pH-dependent association–dissociation equilibrium between monomer and polymers. The KTI lane shows the protein (≈21 kDa) and some weaker bands.
